# Metabolic dysfunction-associated steatotic liver disease

**DOI:** 10.1016/j.clinme.2025.100526

**Published:** 2025-10-25

**Authors:** Catherine Hsu, William Alazawi

**Affiliations:** Queen Mary University of London

**Keywords:** Liver, Metabolic, Community, Lifestyle, Multi-disciplinary

## Abstract

Metabolic dysfunction-associated steatotic liver disease (MASLD), formerly non-alcoholic fatty liver disease (NAFLD), poses a significant challenge to public health. With the rise of obesity, hepatic steatosis now affects around a third of the UK population to varying degrees, frequently coexisting with other cardiometabolic conditions such as type 2 diabetes and ischaemic heart disease. Most patients with MASLD will be diagnosed in the community through investigation of deranged liver function tests or incidentally on abdominal ultrasound. It is therefore important for the general physician to be able to diagnose and risk-stratify MASLD, ensuring appropriate management and onward referral to hepatology for individuals with moderate or high risk of fibrosis.

**Key points**

• MASLD is an increasingly prevalent condition worldwide and coexists with other cardiometabolic conditions.
• There are different phenotypes of MASLD, requiring characterisation through further research.
• The general physician should be confident to diagnose MASLD, give lifestyle advice and risk-stratify for fibrosis.
• Non-invasive tests such as FIB-4 are helpful in assessing the risk of fibrosis and guiding onward referral.
• A number of drugs are in development and health services should be adapted to optimise the delivery of these.

Alt-text: Unlabelled box

## Introduction

Metabolic dysfunction-associated steatotic liver disease (MASLD) is the leading cause of liver disease, affecting up to 30% of the global population at varying degrees of severity.^1^ The nomenclature was changed from ‘non-alcoholic fatty liver disease’ (NAFLD) in 2023 following international consensus acknowledging the importance of reducing stigma, and using inclusive rather than exclusive descriptors.^2^ Given the increasing prevalence of MASLD and its close association with cardiometabolic comorbidities, it is important for the general physician to have a good understanding of this condition.

### Epidemiology

The prevalence of MASLD differs between ethnicities, from 10–15% in Black individuals to 40–45% in Hispanic individuals.^3^^,^^4^ The prevalence among Caucasians and South Asians sits in between, though there is accumulating evidence that South Asians are affected by a more aggressive phenotype.^5^ MASLD affects 60–70% of people with type 2 diabetes and 75% of people with obesity.^6^

Up to 20% of patients with MASLD have a normal body mass index (BMI), a phenotype known as ‘lean MASLD’. This phenotype disproportionately affects those of South Asian ethnicity, and is associated with worse liver and cardiometabolic outcomes than lean controls.^7^^,^^8^ Lean MASLD presents a diagnostic challenge and highlights the importance of developing ethnicity-specific screening criteria and BMI targets.

### Pathogenesis and natural history

MASLD represents a complex interaction between genetic risk and environmental exposure. Risk factors include physical inactivity, social deprivation and a diet high in carbohydrate and saturated fat. The estimated heritability is at least 50%; genome-wide association studies have implicated a number of genetic variants involved in lipid metabolism, including in *PNPLA3* and *TM6SF2*.^9^ In 10–20% of individuals, hepatic steatosis can be complicated by a state of inflammation, lipotoxicity and apoptosis known as metabolic dysfunction-associated steatohepatitis (MASH). This is the precursor to fibrosis, and is an important predictor of adverse liver outcomes including decompensated cirrhosis, liver cancer and need for liver transplantation.^10^ Although viral hepatitis remains the leading cause of liver cancer worldwide currently, the global disease registry shows that the annual change in liver cancer incidence arising from MASLD is greater than that arising from any other aetiology.^11^

### Diagnosis

MASLD is diagnosed when there is excess hepatic fat in the presence of at least one metabolic comorbidity (Table 1).

**Table 1:** Metabolic comorbidities required to diagnose MASLD in adults (adapted from AASLD).

At least one out of five of the following
BMI ≥25 kg/m^2^ (23 kg/m^2^ Asian ethnicity) OR waist circumference >94 cm (men) >80 cm (women).
Fasting glucose ≥5.6 mmol/L OR 2 hours post-load glucose ≥7.8 mmol/L OR HbA1c ≥39 mmol/L OR T2D diagnosis.
Hypertension
Plasma triglycerides ≥1.7 mmol/L.
HDL-cholesterol ≤1 mmol/L (men), 1.3 mmol/L (women).

Patients are usually asymptomatic until advanced stages, so early diagnosis remains a challenge. Unlike conditions such as type 2 diabetes with a single diagnostic test (HbA1c), MASLD has no equivalent. Liver biopsy is the gold standard, but is neither feasible nor desirable as a first-line investigation.

MASLD should be suspected in those with cardiometabolic risk factors and chronically elevated serum alanine aminotransferase (ALT). It is important to rule out other causes through a detailed medical history (particularly an accurate alcohol history), followed by a liver aetiology screen. An ultrasound scan should be performed to assess hepatic texture, contour, vasculature and features of portal hypertension such as splenomegaly that suggest advanced liver disease. If hepatic steatosis is identified, clinicians should assess the risk of fibrosis using non-invasive tests recommended by the National Institute for Health and Car Excellence (NICE), the European Association for the Study of the Liver (EASL) and the American Association for the Study of Liver Diseases (AASLD). These tests combine blood tests and anthropometrics to generate a risk score for fibrosis, represented as ‘low’, ‘intermediate/indeterminate’ or ‘high’.

Individuals with low risk can remain in the community with regular repeat testing. Individuals with high risk are referred to hepatology. Individuals with indeterminate scores have further testing in the form of a blood test known as ‘enhanced liver fibrosis (ELF)’, levels of which correlate with the risk of fibrosis; or can be referred directly for transient elastography (TE).^12^ TE is well validated and uses ultrasound to estimate the degree of fibrosis (liver stiffness measurement, LSM) and steatosis (controlled attenuation parameter CAP).

Both non-invasive blood tests and FibroScan have their limitations. FIB-4 is less accurate at the extremes of ages and appears less sensitive in those of South Asian ethnicity.^13^ TE can provide falsely elevated stiffness readings during active hepatic inflammation, heavy alcohol consumption, venous congestion and in the presence of liver lesions.

## Management

### Lifestyle intervention

Behavioural and lifestyle modifications form the cornerstone of management. Weight loss of 5–7% can resolve hepatic steatosis; weight loss of 10% can resolve MASH and even fibrosis, although even with intensive programmes, this can be difficult to achieve and maintain.^14^ A Mediterranean diet rich in fibre and lean proteins is associated with improved outcomes;^15^ however, cultural dietary differences and increasing food insecurity may limit patients’ ability to choose healthy diets. Dietary advice and support thus need to be tailored to the individual, and supported by institutional changes in food policy.

Physical activity is recommended (>150 min/week of moderate-intensity or 75 min/week of vigorous-intensity exercise) even for individuals of normal body weight, as exercise has been shown to improve the relatively higher levels of visceral obesity in this cohort (Table 2, Table 3).

**Table 2:** Blood-based non-invasive tests used in MASLD.

Test	Components	Range	Limitations
Fibrosis-4 (FIB-4)	Age AST ALT Platelets	<1.3: low risk of fibrosis ≥1.3: intermediate >2.67: high risk of advanced fibrosis	Less reliable if <35 or >65 years old

NAFLD fibrosis score (NFS)	Age AST ALT Platelet BMI Albumin	<−1.455: F0–F2 −1.455 to 0.675: indeterminate >0.675: F3-F4	May overestimate fibrosis in those of high BMI

AST to platelet ratio index (APRI)	AST Platelets	<0.5: low risk of fibrosis 0.5–1.5: indeterminate >1.5: high risk of fibrosis	Affected by conditions affecting platelet count

Enhanced liver fibrosis (ELF)	Three serum biomarkers	<7.7: low risk 7.7–9.8: intermediate risk >9.8: high risk	Expensive, not always available

**Table 3:** Drugs in phase 3 trials to treat MASH at the time of writing.

Target	Drug name
Glucagon-like peptide-1 receptor (GLP-1R) agonist	Semaglutide
Dual GLP-T1R and glucagon receptor agonist	Survodutide
Fibroblast growth factor agonist	Efruxifermin, pegozafermin
Peroxisome proliferator-activated receptors agonist	Lanifibranor
Thyroid hormone receptor-β agonist	Resmetirom

### Pharmacological

There are currently no licensed pharmacological therapies for MASLD in the UK, though many agents show promise in resolving steatohepatitis and improving advanced fibrosis. These therapies target glucose homeostasis, lipid metabolism, inflammation or fibrosis. Resmetirom, a thyroid hormone receptor-β (THR-β) agonist, leads to MASH resolution and improved fibrosis in up to 30% of patients.^16^ It is licensed in the USA and Europe for MASH with moderate to advanced fibrosis. Semaglutide (a GLP-1 receptor agonist) significantly improves fibrosis and resolves steatohepatitis and is licensed in the USA. Many other drug classes are in advanced-stage clinical trials, as are combinations of these. Retrospective studies have suggested an association between statins and reduced progression to fibrosis in MASLD; however, there is insufficient data to recommend their routine prescribing for this indication alone.^17^ Given that cardiovascular disease is the leading cause of death in patients with MASLD, it is important and indeed safe to prescribe statins according to traditional cardiovascular risk scores in this cohort.^18^

### Future directions

The rapidly evolving treatment landscape will change the way we care for patients with MASLD. Given the wide-ranging effects of these drugs as well as the importance of lifestyle modification, we need to reimagine our services with a strong emphasis on the multidisciplinary team, including dieticians, hepatologists, endocrinologists, psychologists and bariatric surgeons in order to deliver holistic and effective care.

In keeping with the NHS 10-year plan of moving from hospitals to the community, there are a number of screening programmes underway. At-risk populations such as those with high alcohol intake, viral hepatitis or those with known hepatic steatosis are invited for TE in diagnostic centres. Clear protocols will direct non-hepatologists to identify individuals with fibrosis in need of onward referral. Recognising the high prevalence of MASLD within the type 2 diabetes population, PRELUDE-1 is a multi-centre prospective cohort study that embeds FIB-4 into the annual diabetes review (results expected later this year).^19^

We also need to ensure that therapies are efficacious across different cohorts. This will require equal representation of different phenotypes of MASLD and different ethnicities in clinical trials and basic research. MASLD is a heterogeneous and complex disease, and only by improving our understanding of its mechanisms can we move towards more effective diagnostic and therapeutic strategies.

## Funding

This research did not receive any specific grant from funding agencies in the public, commercial or not-for-profit sectors.

## CRediT authorship contribution statement

**Catherine Hsu:** Writing – review & editing, Writing – original draft, Conceptualization. **William Alazawi:** Writing – review & editing, Supervision.

## Declaration of competing interest

The authors declare that they have no known competing financial interests or personal relationships that could have appeared to influence the work reported in this paper.

## References

[1] Miao L., Targher G., Byrne C.D., Cao Y., Zheng M. (2024). Current status and future trends of the global burden of MASLD. Trends Endocrinol Metab.

[2] Rinella M.E., Lazarus V., Ratziu V. (2023). A multi-society Delphi consensus statement on new fatty liver disease nomenclature. Hepatology.

[3] Browning J.D., Szczepaniak L.S., Dobbins R. (2004). Prevalence of hepatic steatosis in an urban population in the United States: impact of ethnicity. Hepatology.

[4] Rich N.E., Oji S., Mufti A.R. (2018). Racial and ethnic disparities in nonalcoholic fatty liver disease prevalence, severity, and outcomes in the United States: a systematic review and meta-analysis. Clin Gastroenterol Hepatol.

[5] Alazawi W., Mathur R., Abeysekera K. (2014). Ethnicity and the diagnosis gap in liver disease: a population-based study. Br J Gen Pract.

[6] Latif S., Ahsan T. (2024). Prevalence of metabolic dysfunction-associated steatotic liver disease (MASLD) in persons with obesity and type 2 diabetes mellitus: a cross-sectional study. Euroasian J Hepatogastroenterol.

[7] Golabi P., Paik J., Fukui N., Locklear C.T., de Avilla L., Younossi Z.M. (2019). Patients with lean nonalcoholic fatty liver disease are metabolically abnormal and have a higher risk for mortality. Clin Diabetes.

[8] Ye Q., Zou B., Yeo Y. (2020). Global prevalence, incidence, and outcomes of non-obese or lean non-alcoholic fatty liver disease: a systematic review and meta-analysis. Lancet Gastroenterol Hepatol.

[9] Eslam M., Valenti L., Romeo S. (2018). Genetics and epigenetics of NAFLD and NASH: clinical impact. J Hepatol.

[10] Lekakis V., Papatheodoridis G. (2024). Natural history of metabolic dysfunction-associated steatotic liver disease. Eur J Intern Med.

[11] Giri S., Ingawale S., Khatana G. (2025). Metabolic cause of cirrhosis is the emerging etiology for primary liver cancer in the Asia-Oceania region: analysis of Global Burden of Disease (GBD) Study 2021. J Gastroenterol Hepatol.

[12] Siddiqui M.S., Yamada G., Vuppalanchi R. (2019). Diagnostic accuracy of noninvasive fibrosis models to detect change in fibrosis stage. Clin Gastroenterol Hepatol.

[13] De Silva S., Li W., Kemos P. (2018). Non-invasive markers of liver fibrosis in fatty liver disease are unreliable in people of South Asian descent. Frontline Gastroenterol.

[14] Zeng J., Fan J., Francque S. (2024). Therapeutic management of metabolic dysfunction associated steatotic liver disease. United Eur Gastroenterol J.

[15] Anania C., Perla F.M., Olivero F., Pacifico L., Chiesa C. (2018). Mediterranean diet and nonalcoholic fatty liver disease. World J Gastroenterol.

[16] Harrison S., Bedossa P., Guy C. (2024). A phase 3, randomized, controlled trial of resmetirom in NASH with liver fibrosis. N Engl J Med.

[17] Schreiner A., Zhang J., Petz C. (2024). Statin prescriptions and progression of advanced fibrosis risk in primary care patients with MASLD. BMJ Open Gastroenterol.

[18] Chan W., Chuah K., Rajaram R., Lim L., Ratnasingam J., Vethakkan S.R. (2023). Metabolic dysfunction-associated steatotic liver disease (MASLD): a State-of-the-art review. J Obes Metab Syndr.

[19] Brindley J., Abeysekera K., Hood G. (2023). Feasibility and acceptability of a primary care liver fibrosis testing pathway centred on the diabetes annual review: PRELUDE1 prospective cohort study protocol. BMJ Open..

